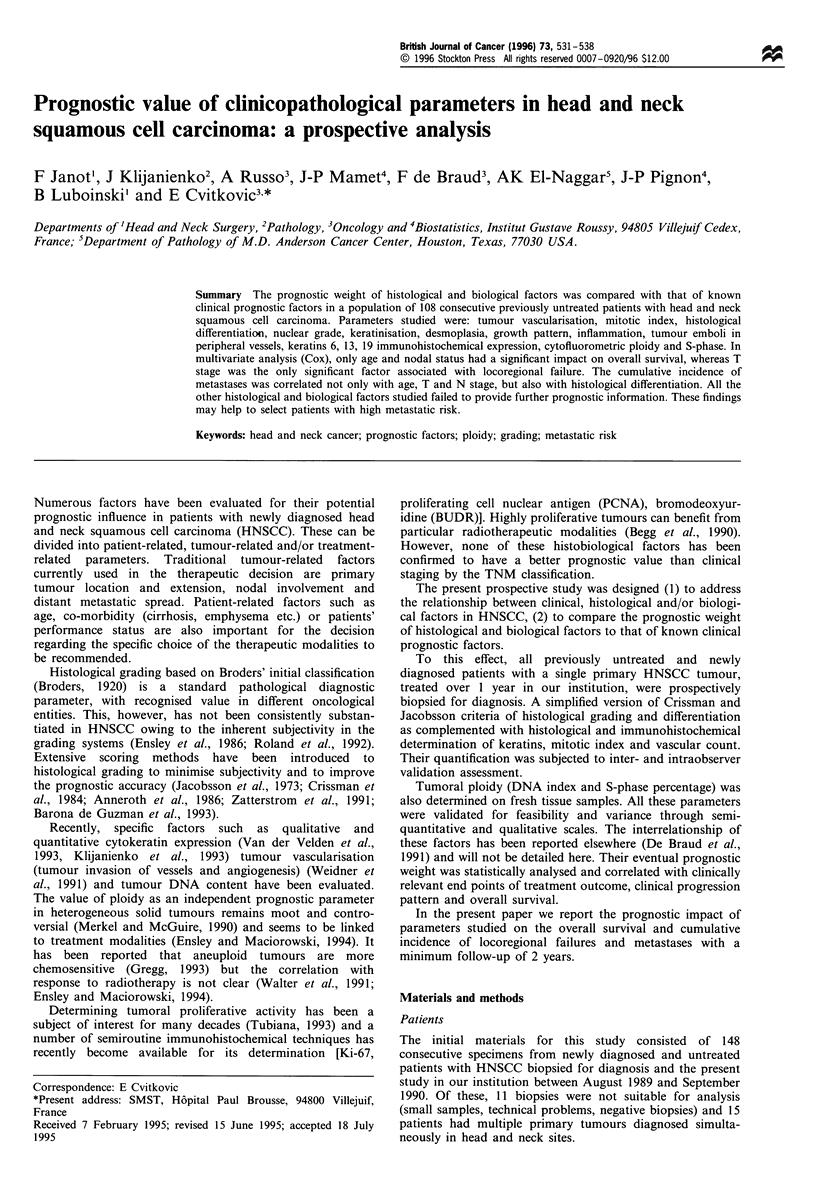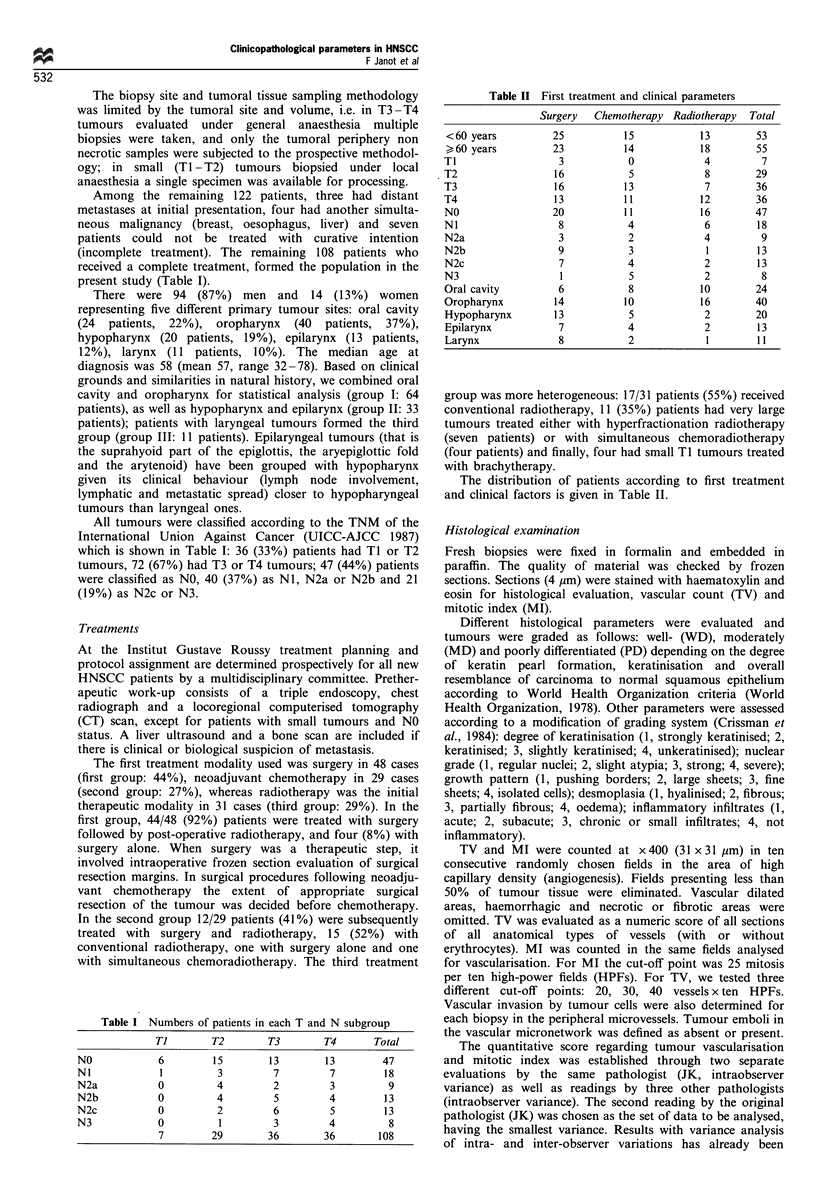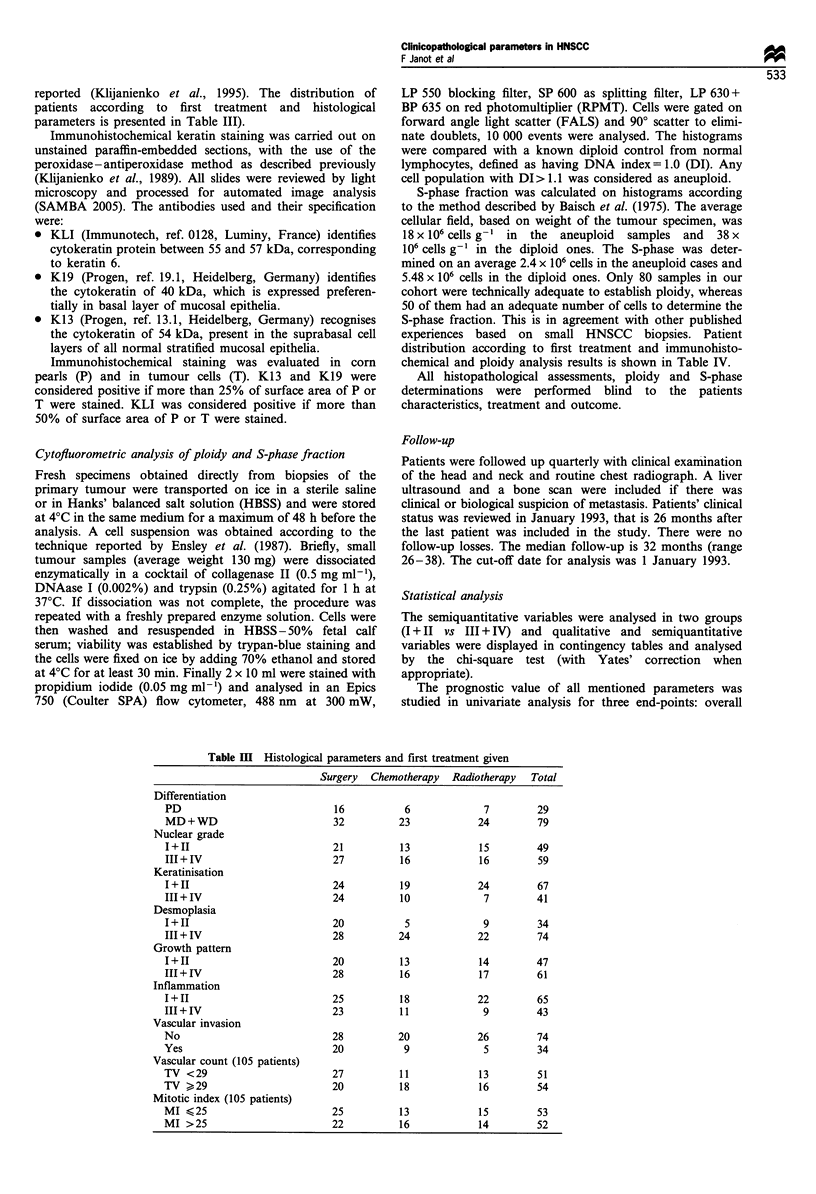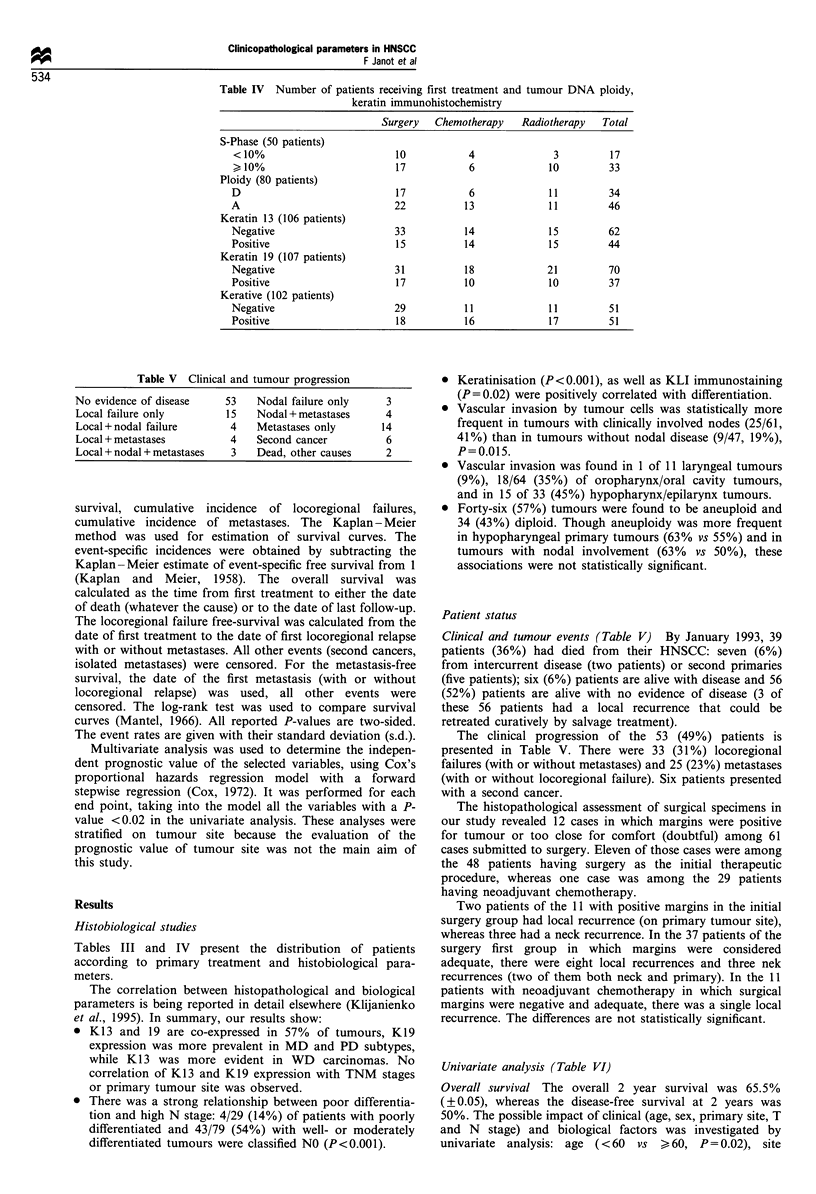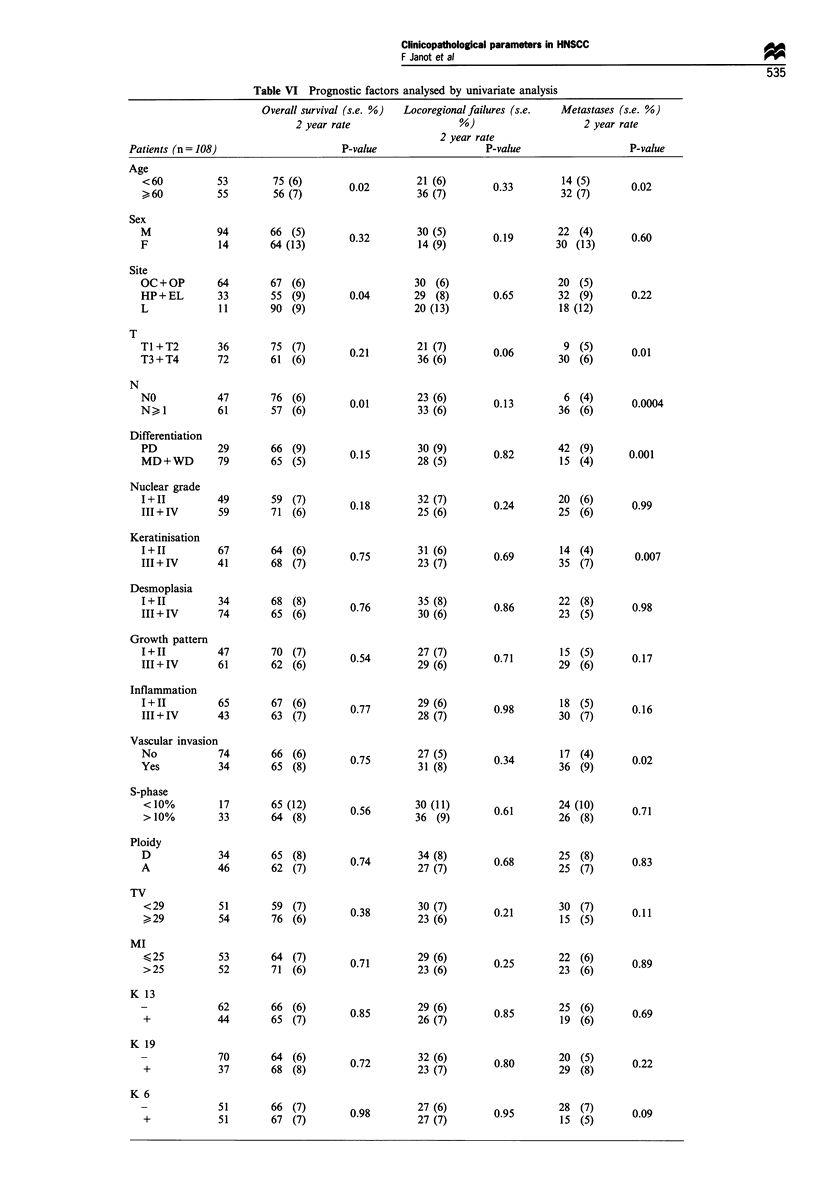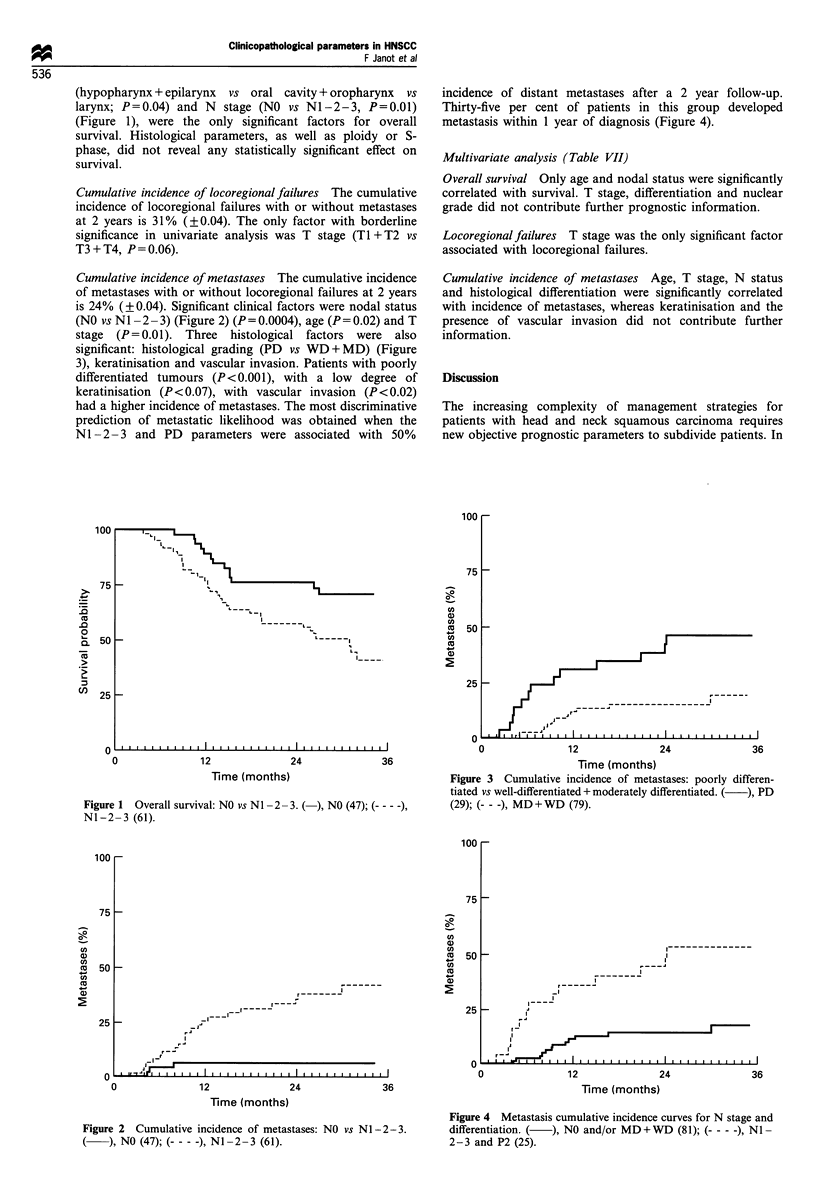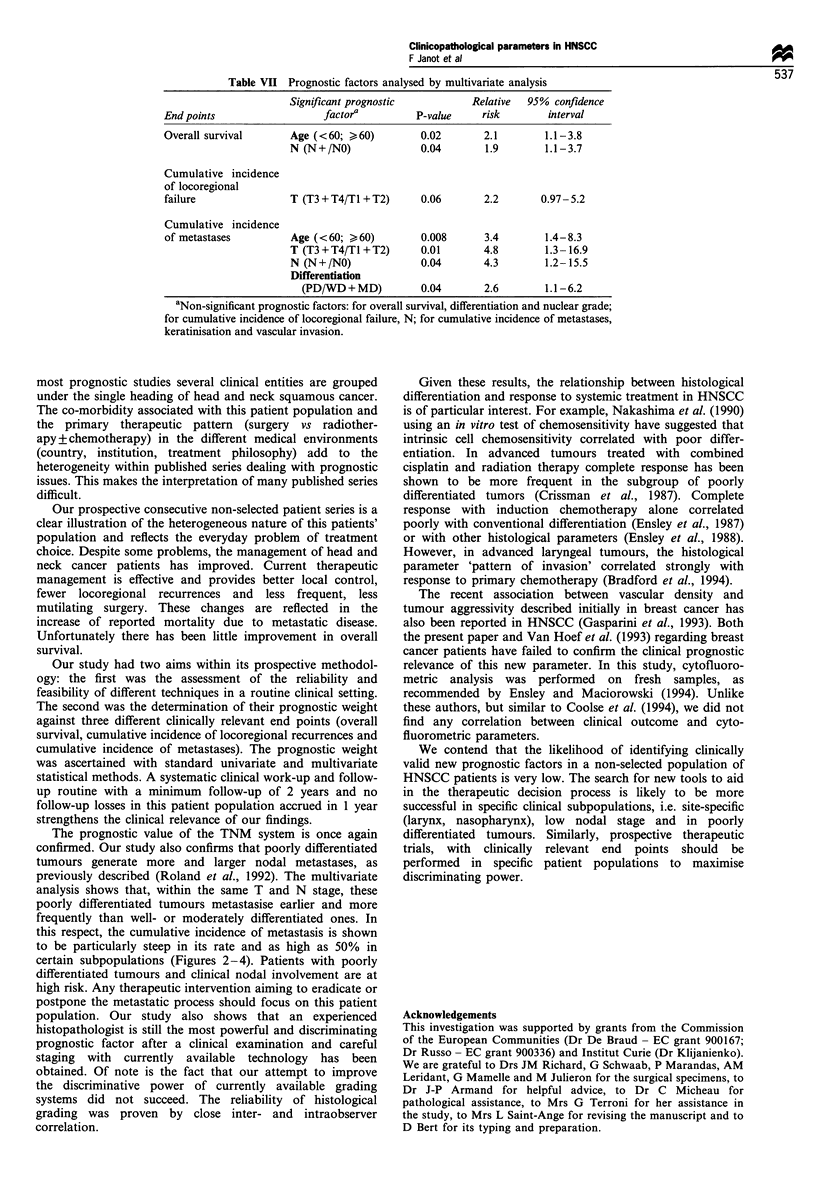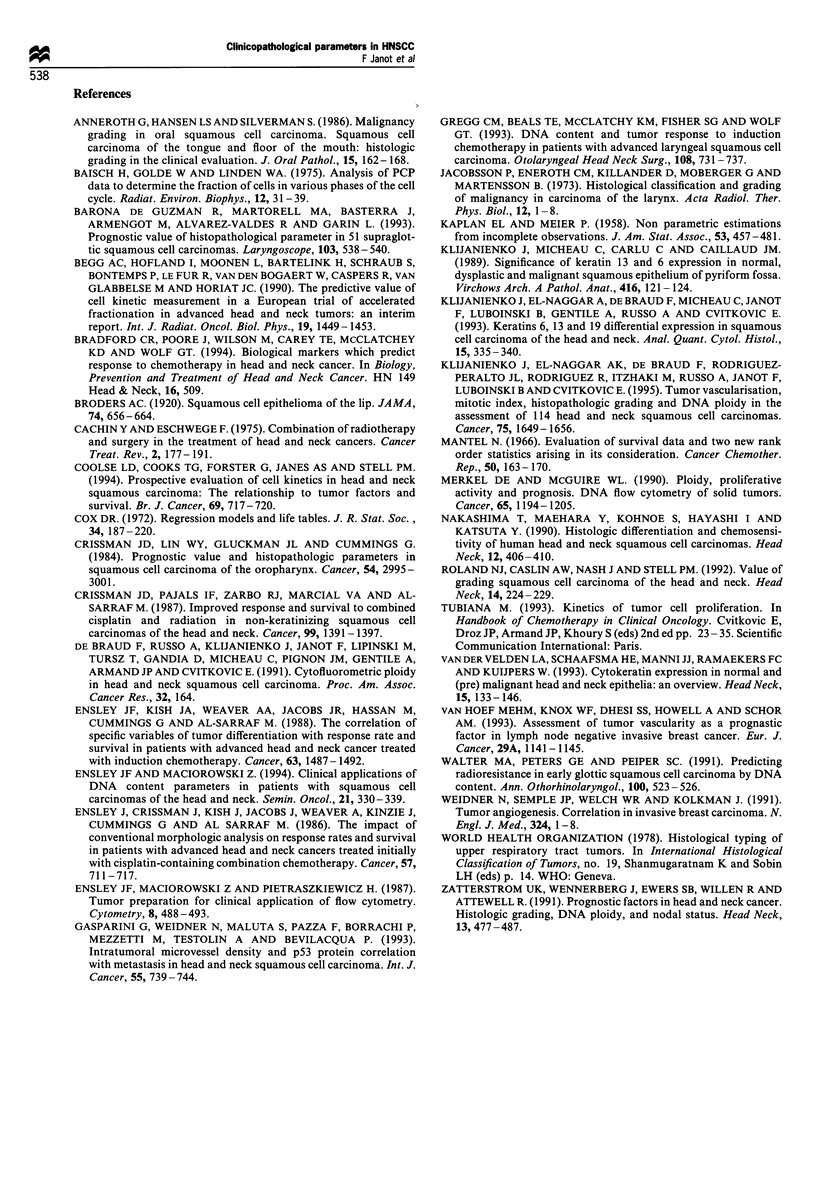# Prognostic value of clinicopathological parameters in head and neck squamous cell carcinoma: a prospective analysis.

**DOI:** 10.1038/bjc.1996.92

**Published:** 1996-02

**Authors:** F. Janot, J. Klijanienko, A. Russo, J. P. Mamet, F. de Braud, A. K. El-Naggar, J. P. Pignon, B. Luboinski, E. Cvitkovic

**Affiliations:** Department of Head and Neck Surgery, Institut Gustave Roussy, Villejuif, France.

## Abstract

The prognostic weight of histological and biological factors was compared with that of known clinical prognostic factors in a population of 108 consecutive previously untreated patients with head and neck squamous cell carcinoma. Parameters studied were: tumour vascularisation, mitotic index, histological differentiation, nuclear grade, keratinisation, desmoplasia, growth pattern, inflammation, tumour emboli in peripheral vessels, keratins 6, 13, 19 immunohistochemical expression, cytofluorometric ploidy and S-phase. In multivariate analysis (Cox), only age and nodal status had a significant impact on the overall survival, whereas T stage was the only significant factor associated with locoregional failure. The cumulative incidence of metastases was correlated not only with age, T and N stage, but also with histological differentiation. All the other histological and biological factors studied failed to provide further prognostic information. These findings may help to select patients with high metastatic risk.


British Journal of Cancer (1996) 73, 531-538
© 1996 Stockton Press All rights reserved 0007-0920/96 $12.00

### Prognostic value of clinicopathological parameters in head and neck squamous cell carcinoma: a prospective analysis

F Janot[1], J Klijanienko[2], A Russo[3], J-P Mamet[4], F de Braud[3], AK El-Naggar[5], J-P Pignon[4], B Luboinski[1] and E Cvitkovic[3,*]

*Departments of [1]Head and Neck Surgery, [2]Pathology, [3]Oncology and [4]Biostatistics, Institut Gustave Roussy, 94805 Villejuif Cedex, France; [5]Department of Pathology of M.D. Anderson Cancer Center, Houston, Texas, 77030 USA.*

**Summary** The prognostic weight of histological and biological factors was compared with that of known clinical prognostic factors in a population of 108 consecutive previously untreated patients with head and neck squamous cell carcinoma. Parameters studied were: tumour vascularisation, mitotic index, histological differentiation, nuclear grade, keratinisation, desmoplasia, growth pattern, inflammation, tumour emboli in peripheral vessels, keratins 6, 13, 19 immunohistochemical expression, cytofluorometric ploidy and S-phase. In multivariate analysis (Cox), only age and nodal status had a significant impact on overall survival, whereas T stage was the only significant factor associated with locoregional failure. The cumulative incidence of metastases was correlated not only with age, T and N stage, but also with histological differentiation. All the other histological and biological factors studied failed to provide further prognostic information. These findings may help to select patients with high metastatic risk.

**Keywords:** head and neck cancer; prognostic factors; ploidy; grading; metastatic risk

Numerous factors have been evaluated for their potential prognostic influence in patients with newly diagnosed head and neck squamous cell carcinoma (HNSCC). These can be divided into patient-related, tumour-related and/or treatment-related parameters. Traditional tumour-related factors currently used in the therapeutic decision are primary tumour location and extension, nodal involvement and distant metastatic spread. Patient-related factors such as age, co-morbidity (cirrhosis, emphysema etc.) or patients' performance status are also important for the decision regarding the specific choice of the therapeutic modalities to be recommended.

Histological grading based on Broders' initial classification (Broders, 1920) is a standard pathological diagnostic parameter, with recognised value in different oncological entities. This, however, has not been consistently substantiated in HNSCC owing to the inherent subjectivity in the grading systems (Ensley *et al.*, 1986; Roland *et al.*, 1992). Extensive scoring methods have been introduced to histological grading to minimise subjectivity and to improve the prognostic accuracy (Jacobsson *et al.*, 1973; Crissman *et al.*, 1984; Anneroth *et al.*, 1986; Zatterstrom *et al.*, 1991; Barona de Guzman *et al.*, 1993).

Recently, specific factors such as qualitative and quantitative cytokeratin expression (Van der Velden *et al.*, 1993, Klijanienko *et al.*, 1993) tumour vascularisation (tumour invasion of vessels and angiogenesis) (Weidner *et al.*, 1991) and tumour DNA content have been evaluated. The value of ploidy as an independent prognostic parameter in heterogeneous solid tumours remains moot and controversial (Merkel and McGuire, 1990) and seems to be linked to treatment modalities (Ensley and Maciorowski, 1994). It has been reported that aneuploid tumours are more chemosensitive (Gregg, 1993) but the correlation with response to radiotherapy is not clear (Walter *et al.*, 1991; Ensley and Maciorowski, 1994).

Determining tumoral proliferative activity has been a subject of interest for many decades (Tubiana, 1993) and a number of semiroutine immunohistochemical techniques has recently become available for its determination [Ki-67, proliferating cell nuclear antigen (PCNA), bromodeoxyuridine (BUDR)]. Highly proliferative tumours can benefit from particular radiotherapeutic modalities (Begg *et al.*, 1990). However, none of these histobiological factors has been confirmed to have a better prognostic value than clinical staging by the TNM classification.

The present prospective study was designed (1) to address the relationship between clinical, histological and/or biological factors in HNSCC, (2) to compare the prognostic weight of histological and biological factors to that of known clinical prognostic factors.

To this effect, all previously untreated and newly diagnosed patients with a single primary HNSCC tumour, treated over 1 year in our institution, were prospectively biopsied for diagnosis. A simplified version of Crissman and Jacobsson criteria of histological grading and differentiation as complemented with histological and immunohistochemical determination of keratins, mitotic index and vascular count. Their quantification was subjected to inter- and intraobserver validation assessment.

Tumoral ploidy (DNA index and S-phase percentage) was also determined on fresh tissue samples. All these parameters were validated for feasibility and variance through semi-quantitative and qualitative scales. The interrelationship of these factors has been reported elsewhere (De Braud *et al.*, 1991) and will not be detailed here. Their eventual prognostic weight was statistically analysed and correlated with clinically relevant end points of treatment outcome, clinical progression pattern and overall survival.

In the present paper we report the prognostic impact of parameters studied on the overall survival and cumulative incidence of locoregional failures and metastases with a minimum follow-up of 2 years.

### Materials and methods

### *Patients*

The initial materials for this study consisted of 148 consecutive specimens from newly diagnosed and untreated patients with HNSCC biopsied for diagnosis and the present study in our institution between August 1989 and September 1990. Of these, 11 biopsies were not suitable for analysis (small samples, technical problems, negative biopsies) and 15 patients had multiple primary tumours diagnosed simultaneously in head and neck sites.

Correspondence: E Cvitkovic
*Present address: SMST, Hôpital Paul Brousse, 94800 Villejuif, France
Received 7 February 1995; revised 15 June 1995; accepted 18 July 1995

The biopsy site and tumoral tissue sampling methodology was limited by the tumoral site and volume, i.e. in T3–T4 tumours evaluated under general anaesthesia multiple biopsies were taken, and only the tumoral periphery non necrotic samples were subjected to the prospective methodology; in small (T1–T2) tumours biopsied under local anaesthesia a single specimen was available for processing.

Among the remaining 122 patients, three had distant metastases at initial presentation, four had another simultaneous malignancy (breast, oesophagus, liver) and seven patients could not be treated with curative intention (incomplete treatment). The remaining 108 patients who received a complete treatment, formed the population in the present study (Table I).

There were 94 (87%) men and 14 (13%) women representing five different primary tumour sites: oral cavity (24 patients, 22%), oropharynx (40 patients, 37%), hypopharynx (20 patients, 19%), epilarynx (13 patients, 12%), larynx (11 patients, 10%). The median age at diagnosis was 58 (mean 57, range 32–78). Based on clinical grounds and similarities in natural history, we combined oral cavity and oropharynx for statistical analysis (group I: 64 patients), as well as hypopharynx and epilarynx (group II: 33 patients); patients with laryngeal tumours formed the third group (group III: 11 patients). Epilaryngeal tumours (that is the suprahyoid part of the epiglottis, the aryepiglottic fold and the arytenoid) have been grouped with hypopharynx given its clinical behaviour (lymph node involvement, lymphatic and metastatic spread) closer to hypopharyngeal tumours than laryngeal ones.

All tumours were classified according to the TNM of the International Union Against Cancer (UICC-AJCC 1987) which is shown in Table I: 36 (33%) patients had T1 or T2 tumours, 72 (67%) had T3 or T4 tumours; 47 (44%) patients were classified as N0, 40 (37%) as N1, N2a or N2b and 21 (19%) as N2c or N3.

*Treatments*

At the Institut Gustave Roussy treatment planning and protocol assignment are determined prospectively for all new HNSCC patients by a multidisciplinary committee. Pretherapeutic work-up consists of a triple endoscopy, chest radiograph and a locoregional computerised tomography (CT) scan, except for patients with small tumours and N0 status. A liver ultrasound and a bone scan are included if there is clinical or biological suspicion of metastasis.

The first treatment modality used was surgery in 48 cases (first group: 44%), neoadjuvant chemotherapy in 29 cases (second group: 27%), whereas radiotherapy was the initial therapeutic modality in 31 cases (third group: 29%). In the first group, 44/48 (92%) patients were treated with surgery followed by post-operative radiotherapy, and four (8%) with surgery alone. When surgery was a therapeutic step, it involved intraoperative frozen section evaluation of surgical resection margins. In surgical procedures following neoadjuvant chemotherapy the extent of appropriate surgical resection of the tumour was decided before chemotherapy. In the second group 12/29 patients (41%) were subsequently treated with surgery and radiotherapy, 15 (52%) with conventional radiotherapy, one with surgery alone and one with simultaneous chemoradiotherapy. The third treatment group was more heterogeneous: 17/31 patients (55%) received conventional radiotherapy, 11 (35%) patients had very large tumours treated either with hyperfractionation radiotherapy (seven patients) or with simultaneous chemoradiotherapy (four patients) and finally, four had small T1 tumours treated with brachytherapy.

The distribution of patients according to first treatment and clinical factors is given in Table II.

**Table I** Numbers of patients in each T and N subgroup

| | *T1* | *T2* | *T3* | *T4* | *Total* |
|---|---|---|---|---|---|
| N0 | 6 | 15 | 13 | 13 | 47 |
| N1 | 1 | 3 | 7 | 7 | 18 |
| N2a | 0 | 4 | 2 | 3 | 9 |
| N2b | 0 | 4 | 5 | 4 | 13 |
| N2c | 0 | 2 | 6 | 5 | 13 |
| N3 | 0 | 1 | 3 | 4 | 8 |
| | 7 | 29 | 36 | 36 | 108 |

**Table II** First treatment and clinical parameters

| | *Surgery* | *Chemotherapy* | *Radiotherapy* | *Total* |
|---|---|---|---|---|
| <60 years | 25 | 15 | 13 | 53 |
| ⩾60 years | 23 | 14 | 18 | 55 |
| T1 | 3 | 0 | 4 | 7 |
| T2 | 16 | 5 | 8 | 29 |
| T3 | 16 | 13 | 7 | 36 |
| T4 | 13 | 11 | 12 | 36 |
| N0 | 20 | 11 | 16 | 47 |
| N1 | 8 | 4 | 6 | 18 |
| N2a | 3 | 2 | 4 | 9 |
| N2b | 9 | 3 | 1 | 13 |
| N2c | 7 | 4 | 2 | 13 |
| N3 | 1 | 5 | 2 | 8 |
| Oral cavity | 6 | 8 | 10 | 24 |
| Oropharynx | 14 | 10 | 16 | 40 |
| Hypopharynx | 13 | 5 | 2 | 20 |
| Epilarynx | 7 | 4 | 2 | 13 |
| Larynx | 8 | 2 | 1 | 11 |

*Histological examination*

Fresh biopsies were fixed in formalin and embedded in paraffin. The quality of material was checked by frozen sections. Sections (4 μm) were stained with haematoxylin and eosin for histological evaluation, vascular count (TV) and mitotic index (MI).

Different histological parameters were evaluated and tumours were graded as follows: well- (WD), moderately (MD) and poorly differentiated (PD) depending on the degree of keratin pearl formation, keratinisation and overall resemblance of carcinoma to normal squamous epithelium according to World Health Organization criteria (World Health Organization, 1978). Other parameters were assessed according to a modification of grading system (Crissman *et al*., 1984): degree of keratinisation (1, strongly keratinised; 2, keratinised; 3, slightly keratinised; 4, unkeratinised); nuclear grade (1, regular nuclei; 2, slight atypia; 3, strong; 4, severe); growth pattern (1, pushing borders; 2, large sheets; 3, fine sheets; 4, isolated cells); desmoplasia (1, hyalinised; 2, fibrous; 3, partially fibrous; 4, oedema); inflammatory infiltrates (1, acute; 2, subacute; 3, chronic or small infiltrates; 4, not inflammatory).

TV and MI were counted at $\times 400$ ($31 \times 31$ μm) in ten consecutive randomly chosen fields in the area of high capillary density (angiogenesis). Fields presenting less than 50% of tumour tissue were eliminated. Vascular dilated areas, haemorrhagic and necrotic or fibrotic areas were omitted. TV was evaluated as a numeric score of all sections of all anatomical types of vessels (with or without erythrocytes). MI was counted in the same fields analysed for vascularisation. For MI the cut-off point was 25 mitosis per ten high-power fields (HPFs). For TV, we tested three different cut-off points: 20, 30, 40 vessels × ten HPFs. Vascular invasion by tumour cells were also determined for each biopsy in the peripheral microvessels. Tumour emboli in the vascular micronetwork was defined as absent or present.

The quantitative score regarding tumour vascularisation and mitotic index was established through two separate evaluations by the same pathologist (JK, intraobserver variance) as well as readings by three other pathologists (intraobserver variance). The second reading by the original pathologist (JK) was chosen as the set of data to be analysed, having the smallest variance. Results with variance analysis of intra- and inter-observer variations has already been reported (Klijanienko *et al.*, 1995). The distribution of patients according to first treatment and histological parameters is presented in Table III).

Immunohistochemical keratin staining was carried out on unstained paraffin-embedded sections, with the use of the peroxidase–antiperoxidase method as described previously (Klijanienko *et al.*, 1989). All slides were reviewed by light microscopy and processed for automated image analysis (SAMBA 2005). The antibodies used and their specification were:

- KLI (Immunotech, ref. 0128, Luminy, France) identifies cytokeratin protein between 55 and 57 kDa, corresponding to keratin 6.
- K19 (Progen, ref. 19.1, Heidelberg, Germany) identifies the cytokeratin of 40 kDa, which is expressed preferentially in basal layer of mucosal epithelia.
- K13 (Progen, ref. 13.1, Heidelberg, Germany) recognises the cytokeratin of 54 kDa, present in the suprabasal cell layers of all normal stratified mucosal epithelia.

Immunohistochemical staining was evaluated in corn pearls (P) and in tumour cells (T). K13 and K19 were considered positive if more than 25% of surface area of P or T were stained. KLI was considered positive if more than 50% of surface area of P or T were stained.

### *Cytofluorometric analysis of ploidy and S-phase fraction*

Fresh specimens obtained directly from biopsies of the primary tumour were transported on ice in a sterile saline or in Hanks' balanced salt solution (HBSS) and were stored at 4°C in the same medium for a maximum of 48 h before the analysis. A cell suspension was obtained according to the technique reported by Ensley *et al.* (1987). Briefly, small tumour samples (average weight 130 mg) were dissociated enzymatically in a cocktail of collagenase II (0.5 mg $ml^{-1}$), DNAase I (0.002%) and trypsin (0.25%) agitated for 1 h at 37°C. If dissociation was not complete, the procedure was repeated with a freshly prepared enzyme solution. Cells were then washed and resuspended in HBSS–50% fetal calf serum; viability was established by trypan-blue staining and the cells were fixed on ice by adding 70% ethanol and stored at 4°C for at least 30 min. Finally 2 x 10 ml were stained with propidium iodide (0.05 mg $ml^{-1}$) and analysed in an Epics 750 (Coulter SPA) flow cytometer, 488 nm at 300 mW, LP 550 blocking filter, SP 600 as splitting filter, LP 630 + BP 635 on red photomultiplier (RPMT). Cells were gated on forward angle light scatter (FALS) and 90° scatter to eliminate doublets, 10 000 events were analysed. The histograms were compared with a known diploid control from normal lymphocytes, defined as having DNA index = 1.0 (DI). Any cell population with DI > 1.1 was considered as aneuploid.

S-phase fraction was calculated on histograms according to the method described by Baisch *et al.* (1975). The average cellular field, based on weight of the tumour specimen, was $18 \times 10^6$ cells $g^{-1}$ in the aneuploid samples and $38 \times 10^6$ cells $g^{-1}$ in the diploid ones. The S-phase was determined on an average $2.4 \times 10^6$ cells in the aneuploid cases and $5.48 \times 10^6$ cells in the diploid ones. Only 80 samples in our cohort were technically adequate to establish ploidy, whereas 50 of them had an adequate number of cells to determine the S-phase fraction. This is in agreement with other published experiences based on small HNSCC biopsies. Patient distribution according to first treatment and immunohistochemical and ploidy analysis results is shown in Table IV.

All histopathological assessments, ploidy and S-phase determinations were performed blind to the patients characteristics, treatment and outcome.

### *Follow-up*

Patients were followed up quarterly with clinical examination of the head and neck and routine chest radiograph. A liver ultrasound and a bone scan were included if there was clinical or biological suspicion of metastasis. Patients' clinical status was reviewed in January 1993, that is 26 months after the last patient was included in the study. There were no follow-up losses. The median follow-up is 32 months (range 26–38). The cut-off date for analysis was 1 January 1993.

### *Statistical analysis*

The semiquantitative variables were analysed in two groups (I + II *vs* III + IV) and qualitative and semiquantitative variables were displayed in contingency tables and analysed by the chi-square test (with Yates' correction when appropriate).

The prognostic value of all mentioned parameters was studied in univariate analysis for three end-points: overall

**Table III** Histological parameters and first treatment given

| | *Surgery* | *Chemotherapy* | *Radiotherapy* | *Total* |
|---|---|---|---|---|
| Differentiation | | | | |
| PD | 16 | 6 | 7 | 29 |
| MD + WD | 32 | 23 | 24 | 79 |
| Nuclear grade | | | | |
| I + II | 21 | 13 | 15 | 49 |
| III + IV | 27 | 16 | 16 | 59 |
| Keratinisation | | | | |
| I + II | 24 | 19 | 24 | 67 |
| III + IV | 24 | 10 | 7 | 41 |
| Desmoplasia | | | | |
| I + II | 20 | 5 | 9 | 34 |
| III + IV | 28 | 24 | 22 | 74 |
| Growth pattern | | | | |
| I + II | 20 | 13 | 14 | 47 |
| III + IV | 28 | 16 | 17 | 61 |
| Inflammation | | | | |
| I + II | 25 | 18 | 22 | 65 |
| III + IV | 23 | 11 | 9 | 43 |
| Vascular invasion | | | | |
| No | 28 | 20 | 26 | 74 |
| Yes | 20 | 9 | 5 | 34 |
| Vascular count (105 patients) | | | | |
| TV <29 | 27 | 11 | 13 | 51 |
| TV ⩾29 | 20 | 18 | 16 | 54 |
| Mitotic index (105 patients) | | | | |
| MI ⩽25 | 25 | 13 | 15 | 53 |
| MI >25 | 22 | 16 | 14 | 52 |

Table IV Number of patients receiving first treatment and tumour DNA ploidy, keratin immunohistochemistry

| | *Surgery* | *Chemotherapy* | *Radiotherapy* | *Total* |
|---|---|---|---|---|
| S-Phase (50 patients) | | | | |
| <10% | 10 | 4 | 3 | 17 |
| ⩾10% | 17 | 6 | 10 | 33 |
| Ploidy (80 patients) | | | | |
| D | 17 | 6 | 11 | 34 |
| A | 22 | 13 | 11 | 46 |
| Keratin 13 (106 patients) | | | | |
| Negative | 33 | 14 | 15 | 62 |
| Positive | 15 | 14 | 15 | 44 |
| Keratin 19 (107 patients) | | | | |
| Negative | 31 | 18 | 21 | 70 |
| Positive | 17 | 10 | 10 | 37 |
| Kerative (102 patients) | | | | |
| Negative | 29 | 11 | 11 | 51 |
| Positive | 18 | 16 | 17 | 51 |

Table V Clinical and tumour progression

| | | | |
|---|---|---|---|
| No evidence of disease | 53 | Nodal failure only | 3 |
| Local failure only | 15 | Nodal + metastases | 4 |
| Local + nodal failure | 4 | Metastases only | 14 |
| Local + metastases | 4 | Second cancer | 6 |
| Local + nodal + metastases | 3 | Dead, other causes | 2 |

survival, cumulative incidence of locoregional failures, cumulative incidence of metastases. The Kaplan–Meier method was used for estimation of survival curves. The event-specific incidences were obtained by subtracting the Kaplan–Meier estimate of event-specific free survival from 1 (Kaplan and Meier, 1958). The overall survival was calculated as the time from first treatment to either the date of death (whatever the cause) or to the date of last follow-up. The locoregional failure free-survival was calculated from the date of first treatment to the date of first locoregional relapse with or without metastases. All other events (second cancers, isolated metastases) were censored. For the metastasis-free survival, the date of the first metastasis (with or without locoregional relapse) was used, all other events were censored. The log-rank test was used to compare survival curves (Mantel, 1966). All reported *P*-values are two-sided. The event rates are given with their standard deviation (s.d.).

Multivariate analysis was used to determine the independent prognostic value of the selected variables, using Cox's proportional hazards regression model with a forward stepwise regression (Cox, 1972). It was performed for each end point, taking into the model all the variables with a *P*-value $<0.02$ in the univariate analysis. These analyses were stratified on tumour site because the evaluation of the prognostic value of tumour site was not the main aim of this study.

### Results

### *Histobiological studies*

Tables III and IV present the distribution of patients according to primary treatment and histobiological parameters.

The correlation between histopathological and biological parameters is being reported in detail elsewhere (Klijanienko *et al*., 1995). In summary, our results show:

- K13 and 19 are co-expressed in 57% of tumours, K19 expression was more prevalent in MD and PD subtypes, while K13 was more evident in WD carcinomas. No correlation of K13 and K19 expression with TNM stages or primary tumour site was observed.
- There was a strong relationship between poor differentiation and high N stage: 4/29 (14%) of patients with poorly differentiated and 43/79 (54%) with well- or moderately differentiated tumours were classified N0 ($P<0.001$).
- Keratinisation ($P<0.001$), as well as KLI immunostaining ($P=0.02$) were positively correlated with differentiation.
- Vascular invasion by tumour cells was statistically more frequent in tumours with clinically involved nodes (25/61, 41%) than in tumours without nodal disease (9/47, 19%), $P=0.015$.
- Vascular invasion was found in 1 of 11 laryngeal tumours (9%), 18/64 (35%) of oropharynx/oral cavity tumours, and in 15 of 33 (45%) hypopharynx/epilarynx tumours.
- Forty-six (57%) tumours were found to be aneuploid and 34 (43%) diploid. Though aneuploidy was more frequent in hypopharyngeal primary tumours (63% *vs* 55%) and in tumours with nodal involvement (63% *vs* 50%), these associations were not statistically significant.

### *Patient status*

*Clinical and tumour events (Table V)* By January 1993, 39 patients (36%) had died from their HNSCC: seven (6%) from intercurrent disease (two patients) or second primaries (five patients); six (6%) patients are alive with disease and 56 (52%) patients are alive with no evidence of disease (3 of these 56 patients had a local recurrence that could be retreated curatively by salvage treatment).

The clinical progression of the 53 (49%) patients is presented in Table V. There were 33 (31%) locoregional failures (with or without metastases) and 25 (23%) metastases (with or without locoregional failure). Six patients presented with a second cancer.

The histopathological assessment of surgical specimens in our study revealed 12 cases in which margins were positive for tumour or too close for comfort (doubtful) among 61 cases submitted to surgery. Eleven of those cases were among the 48 patients having surgery as the initial therapeutic procedure, whereas one case was among the 29 patients having neoadjuvant chemotherapy.

Two patients of the 11 with positive margins in the initial surgery group had local recurrence (on primary tumour site), whereas three had a neck recurrence. In the 37 patients of the surgery first group in which margins were considered adequate, there were eight local recurrences and three nek recurrences (two of them both neck and primary). In the 11 patients with neoadjuvant chemotherapy in which surgical margins were negative and adequate, there was a single local recurrence. The differences are not statistically significant.

### *Univariate analysis (Table VI)*

*Overall survival* The overall 2 year survival was 65.5% ($\pm0.05$), whereas the disease-free survival at 2 years was 50%. The possible impact of clinical (age, sex, primary site, T and N stage) and biological factors was investigated by univariate analysis: age ($<60$ *vs* $\geqslant60$, $P=0.02$), site

**Table VI** Prognostic factors analysed by univariate analysis

| *Patients (n = 108)* | | *Overall survival (s.e. %) 2 year rate* | P-*value* | *Locoregional failures (s.e. %) 2 year rate* | P-*value* | *Metastases (s.e. %) 2 year rate* | P-*value* |
|---|---|---|---|---|---|---|---|
| Age | | | | | | | |
| <60 | 53 | 75 (6) | 0.02 | 21 (6) | 0.33 | 14 (5) | 0.02 |
| ⩾60 | 55 | 56 (7) | | 36 (7) | | 32 (7) | |
| Sex | | | | | | | |
| M | 94 | 66 (5) | 0.32 | 30 (5) | 0.19 | 22 (4) | 0.60 |
| F | 14 | 64 (13) | | 14 (9) | | 30 (13) | |
| Site | | | | | | | |
| OC+OP | 64 | 67 (6) | 0.04 | 30 (6) | 0.65 | 20 (5) | 0.22 |
| HP+EL | 33 | 55 (9) | | 29 (8) | | 32 (9) | |
| L | 11 | 90 (9) | | 20 (13) | | 18 (12) | |
| T | | | | | | | |
| T1+T2 | 36 | 75 (7) | 0.21 | 21 (7) | 0.06 | 9 (5) | 0.01 |
| T3+T4 | 72 | 61 (6) | | 36 (6) | | 30 (6) | |
| N | | | | | | | |
| N0 | 47 | 76 (6) | 0.01 | 23 (6) | 0.13 | 6 (4) | 0.0004 |
| N⩾1 | 61 | 57 (6) | | 33 (6) | | 36 (6) | |
| Differentiation | | | | | | | |
| PD | 29 | 66 (9) | 0.15 | 30 (9) | 0.82 | 42 (9) | 0.001 |
| MD+WD | 79 | 65 (5) | | 28 (5) | | 15 (4) | |
| Nuclear grade | | | | | | | |
| I+II | 49 | 59 (7) | 0.18 | 32 (7) | 0.24 | 20 (6) | 0.99 |
| III+IV | 59 | 71 (6) | | 25 (6) | | 25 (6) | |
| Keratinisation | | | | | | | |
| I+II | 67 | 64 (6) | 0.75 | 31 (6) | 0.69 | 14 (4) | 0.007 |
| III+IV | 41 | 68 (7) | | 23 (7) | | 35 (7) | |
| Desmoplasia | | | | | | | |
| I+II | 34 | 68 (8) | 0.76 | 35 (8) | 0.86 | 22 (8) | 0.98 |
| III+IV | 74 | 65 (6) | | 30 (6) | | 23 (5) | |
| Growth pattern | | | | | | | |
| I+II | 47 | 70 (7) | 0.54 | 27 (7) | 0.71 | 15 (5) | 0.17 |
| III+IV | 61 | 62 (6) | | 29 (6) | | 29 (6) | |
| Inflammation | | | | | | | |
| I+II | 65 | 67 (6) | 0.77 | 29 (6) | 0.98 | 18 (5) | 0.16 |
| III+IV | 43 | 63 (7) | | 28 (7) | | 30 (7) | |
| Vascular invasion | | | | | | | |
| No | 74 | 66 (6) | 0.75 | 27 (5) | 0.34 | 17 (4) | 0.02 |
| Yes | 34 | 65 (8) | | 31 (8) | | 36 (9) | |
| S-phase | | | | | | | |
| <10% | 17 | 65 (12) | 0.56 | 30 (11) | 0.61 | 24 (10) | 0.71 |
| >10% | 33 | 64 (8) | | 36 (9) | | 26 (8) | |
| Ploidy | | | | | | | |
| D | 34 | 65 (8) | 0.74 | 34 (8) | 0.68 | 25 (8) | 0.83 |
| A | 46 | 62 (7) | | 27 (7) | | 25 (7) | |
| TV | | | | | | | |
| <29 | 51 | 59 (7) | 0.38 | 30 (7) | 0.21 | 30 (7) | 0.11 |
| ⩾29 | 54 | 76 (6) | | 23 (6) | | 15 (5) | |
| MI | | | | | | | |
| ⩽25 | 53 | 64 (7) | 0.71 | 29 (6) | 0.25 | 22 (6) | 0.89 |
| >25 | 52 | 71 (6) | | 23 (6) | | 23 (6) | |
| K 13 | | | | | | | |
| – | 62 | 66 (6) | 0.85 | 29 (6) | 0.85 | 25 (6) | 0.69 |
| + | 44 | 65 (7) | | 26 (7) | | 19 (6) | |
| K 19 | | | | | | | |
| – | 70 | 64 (6) | 0.72 | 32 (6) | 0.80 | 20 (5) | 0.22 |
| + | 37 | 68 (8) | | 23 (7) | | 29 (8) | |
| K 6 | | | | | | | |
| – | 51 | 66 (7) | 0.98 | 27 (6) | 0.95 | 28 (7) | 0.09 |
| + | 51 | 67 (7) | | 27 (7) | | 15 (5) | |

(hypopharynx + epilarynx *vs* oral cavity + oropharynx *vs* larynx; $P=0.04$) and N stage (N0 *vs* N1-2-3, $P=0.01$) (Figure 1), were the only significant factors for overall survival. Histological parameters, as well as ploidy or S-phase, did not reveal any statistically significant effect on survival.

*Cumulative incidence of locoregional failures* The cumulative incidence of locoregional failures with or without metastases at 2 years is 31% (±0.04). The only factor with borderline significance in univariate analysis was T stage (T1 + T2 *vs* T3 + T4, $P=0.06$).

*Cumulative incidence of metastases* The cumulative incidence of metastases with or without locoregional failures at 2 years is 24% (±0.04). Significant clinical factors were nodal status (N0 *vs* N1-2-3) (Figure 2) ($P=0.0004$), age ($P=0.02$) and T stage ($P=0.01$). Three histological factors were also significant: histological grading (PD *vs* WD + MD) (Figure 3), keratinisation and vascular invasion. Patients with poorly differentiated tumours ($P<0.001$), with a low degree of keratinisation ($P<0.07$), with vascular invasion ($P<0.02$) had a higher incidence of metastases. The most discriminative prediction of metastatic likelihood was obtained when the N1-2-3 and PD parameters were associated with 50% incidence of distant metastases after a 2 year follow-up. Thirty-five per cent of patients in this group developed metastasis within 1 year of diagnosis (Figure 4).

*Multivariate analysis (Table VII)*

*Overall survival* Only age and nodal status were significantly correlated with survival. T stage, differentiation and nuclear grade did not contribute further prognostic information.

*Locoregional failures* T stage was the only significant factor associated with locoregional failures.

*Cumulative incidence of metastases* Age, T stage, N status and histological differentiation were significantly correlated with incidence of metastases, whereas keratinisation and the presence of vascular invasion did not contribute further information.

### Discussion

The increasing complexity of management strategies for patients with head and neck squamous carcinoma requires new objective prognostic parameters to subdivide patients. In

**Figure 1** Overall survival: N0 *vs* N1-2-3. (—), N0 (47); (- - - -), N1-2-3 (61).

**Figure 2** Cumulative incidence of metastases: N0 *vs* N1-2-3. (——), N0 (47); (- - - -), N1-2-3 (61).

**Figure 3** Cumulative incidence of metastases: poorly differentiated *vs* well-differentiated + moderately differentiated. (——), PD (29); (- - -), MD + WD (79).

**Figure 4** Metastasis cumulative incidence curves for N stage and differentiation. (——), N0 and/or MD + WD (81); (- - - -), N1-2-3 and P2 (25).

Table VII Prognostic factors analysed by multivariate analysis

| *End points* | *Significant prognostic factor*[a] | P-*value* | *Relative risk* | *95% confidence interval* |
|---|---|---|---|---|
| Overall survival | **Age** (<60; ⩾60) | 0.02 | 2.1 | 1.1–3.8 |
| | N (N+/N0) | 0.04 | 1.9 | 1.1–3.7 |
| Cumulative incidence of locoregional failure | T (T3+T4/T1+T2) | 0.06 | 2.2 | 0.97–5.2 |
| Cumulative incidence of metastases | **Age** (<60; ⩾60) | 0.008 | 3.4 | 1.4–8.3 |
| | T (T3+T4/T1+T2) | 0.01 | 4.8 | 1.3–16.9 |
| | N (N+/N0) | 0.04 | 4.3 | 1.2–15.5 |
| | **Differentiation** (PD/WD+MD) | 0.04 | 2.6 | 1.1–6.2 |

[a]Non-significant prognostic factors: for overall survival, differentiation and nuclear grade; for cumulative incidence of locoregional failure, N; for cumulative incidence of metastases, keratinisation and vascular invasion.

most prognostic studies several clinical entities are grouped under the single heading of head and neck squamous cancer. The co-morbidity associated with this patient population and the primary therapeutic pattern (surgery *vs* radiotherapy±chemotherapy) in the different medical environments (country, institution, treatment philosophy) add to the heterogeneity within published series dealing with prognostic issues. This makes the interpretation of many published series difficult.

Our prospective consecutive non-selected patient series is a clear illustration of the heterogeneous nature of this patients' population and reflects the everyday problem of treatment choice. Despite some problems, the management of head and neck cancer patients has improved. Current therapeutic management is effective and provides better local control, fewer locoregional recurrences and less frequent, less mutilating surgery. These changes are reflected in the increase of reported mortality due to metastatic disease. Unfortunately there has been little improvement in overall survival.

Our study had two aims within its prospective methodology: the first was the assessment of the reliability and feasibility of different techniques in a routine clinical setting. The second was the determination of their prognostic weight against three different clinically relevant end points (overall survival, cumulative incidence of locoregional recurrences and cumulative incidence of metastases). The prognostic weight was ascertained with standard univariate and multivariate statistical methods. A systematic clinical work-up and follow-up routine with a minimum follow-up of 2 years and no follow-up losses in this patient population accrued in 1 year strengthens the clinical relevance of our findings.

The prognostic value of the TNM system is once again confirmed. Our study also confirms that poorly differentiated tumours generate more and larger nodal metastases, as previously described (Roland *et al.*, 1992). The multivariate analysis shows that, within the same T and N stage, these poorly differentiated tumours metastasise earlier and more frequently than well- or moderately differentiated ones. In this respect, the cumulative incidence of metastasis is shown to be particularly steep in its rate and as high as 50% in certain subpopulations (Figures 2–4). Patients with poorly differentiated tumours and clinical nodal involvement are at high risk. Any therapeutic intervention aiming to eradicate or postpone the metastatic process should focus on this patient population. Our study also shows that an experienced histopathologist is still the most powerful and discriminating prognostic factor after a clinical examination and careful staging with currently available technology has been obtained. Of note is the fact that our attempt to improve the discriminative power of currently available grading systems did not succeed. The reliability of histological grading was proven by close inter- and intraobserver correlation.

Given these results, the relationship between histological differentiation and response to systemic treatment in HNSCC is of particular interest. For example, Nakashima *et al.* (1990) using an *in vitro* test of chemosensitivity have suggested that intrinsic cell chemosensitivity correlated with poor differentiation. In advanced tumours treated with combined cisplatin and radiation therapy complete response has been shown to be more frequent in the subgroup of poorly differentiated tumors (Crissman *et al.*, 1987). Complete response with induction chemotherapy alone correlated poorly with conventional differentiation (Ensley *et al.*, 1987) or with other histological parameters (Ensley *et al.*, 1988). However, in advanced laryngeal tumours, the histological parameter 'pattern of invasion' correlated strongly with response to primary chemotherapy (Bradford *et al.*, 1994).

The recent association between vascular density and tumour aggressivity described initially in breast cancer has also been reported in HNSCC (Gasparini *et al.*, 1993). Both the present paper and Van Hoef *et al.* (1993) regarding breast cancer patients have failed to confirm the clinical prognostic relevance of this new parameter. In this study, cytofluorometric analysis was performed on fresh samples, as recommended by Ensley and Maciorowski (1994). Unlike these authors, but similar to Coolse *et al.* (1994), we did not find any correlation between clinical outcome and cytofluorometric parameters.

We contend that the likelihood of identifying clinically valid new prognostic factors in a non-selected population of HNSCC patients is very low. The search for new tools to aid in the therapeutic decision process is likely to be more successful in specific clinical subpopulations, i.e. site-specific (larynx, nasopharynx), low nodal stage and in poorly differentiated tumours. Similarly, prospective therapeutic trials, with clinically relevant end points should be performed in specific patient populations to maximise discriminating power.

**Acknowledgements**

This investigation was supported by grants from the Commission of the European Communities (Dr De Braud – EC grant 900167; Dr Russo – EC grant 900336) and Institut Curie (Dr Klijanienko). We are grateful to Drs JM Richard, G Schwaab, P Marandas, AM Leridant, G Mamelle and M Julieron for the surgical specimens, to Dr J-P Armand for helpful advice, to Dr C Micheau for pathological assistance, to Mrs G Terroni for her assistance in the study, to Mrs L Saint-Ange for revising the manuscript and to D Bert for its typing and preparation.

### References

ANNEROTH G, HANSEN LS AND SILVERMAN S. (1986). Malignancy grading in oral squamous cell carcinoma. Squamous cell carcinoma of the tongue and floor of the mouth: histologic grading in the clinical evaluation. *J. Oral Pathol.*, **15**, 162–168.

BAISCH H, GOLDE W AND LINDEN WA. (1975). Analysis of PCP data to determine the fraction of cells in various phases of the cell cycle. *Radiat. Environ. Biophys.*, **12**, 31–39.

BARONA DE GUZMAN R, MARTORELL MA, BASTERRA J, ARMENGOT M, ALVAREZ-VALDES R AND GARIN L. (1993). Prognostic value of histopathological parameter in 51 supraglottic squamous cell carcinomas. *Laryngoscope*, **103**, 538–540.

BEGG AC, HOFLAND I, MOONEN L, BARTELINK H, SCHRAUB S, BONTEMPS P, LE FUR R, VAN DEN BOGAERT W, CASPERS R, VAN GLABBELSE M AND HORIAT JC. (1990). The predictive value of cell kinetic measurement in a European trial of accelerated fractionation in advanced head and neck tumors: an interim report. *Int. J. Radiat. Oncol. Biol. Phys.*, **19**, 1449–1453.

BRADFORD CR, POORE J, WILSON M, CAREY TE, MCCLATCHEY KD AND WOLF GT. (1994). Biological markers which predict response to chemotherapy in head and neck cancer. In *Biology, Prevention and Treatment of Head and Neck Cancer*. HN 149 Head & Neck, **16**, 509.

BRODERS AC. (1920). Squamous cell epithelioma of the lip. *JAMA*, **74**, 656–664.

CACHIN Y AND ESCHWEGE F. (1975). Combination of radiotherapy and surgery in the treatment of head and neck cancers. *Cancer Treat. Rev.*, **2**, 177–191.

COOLSE LD, COOKS TG, FORSTER G, JANES AS AND STELL PM. (1994). Prospective evaluation of cell kinetics in head and neck squamous carcinoma: The relationship to tumor factors and survival. *Br. J. Cancer*, **69**, 717–720.

COX DR. (1972). Regression models and life tables. *J. R. Stat. Soc.*, **34**, 187–220.

CRISSMAN JD, LIN WY, GLUCKMAN JL AND CUMMINGS G. (1984). Prognostic value and histopathologic parameters in squamous cell carcinoma of the oropharynx. *Cancer*, **54**, 2995–3001.

CRISSMAN JD, PAJALS IF, ZARBO RJ, MARCIAL VA AND AL-SARRAF M. (1987). Improved response and survival to combined cisplatin and radiation in non-keratinizing squamous cell carcinomas of the head and neck. *Cancer*, **99**, 1391–1397.

DE BRAUD F, RUSSO A, KLIJANIENKO J, JANOT F, LIPINSKI M, TURSZ T, GANDIA D, MICHEAU C, PIGNON JM, GENTILE A, ARMAND JP AND CVITKOVIC E. (1991). Cytofluorometric ploidy in head and neck squamous cell carcinoma. *Proc. Am. Assoc. Cancer Res.*, **32**, 164.

ENSLEY JF, KISH JA, WEAVER AA, JACOBS JR, HASSAN M, CUMMINGS G AND AL-SARRAF M. (1988). The correlation of specific variables of tumor differentiation with response rate and survival in patients with advanced head and neck cancer treated with induction chemotherapy. *Cancer*, **63**, 1487–1492.

ENSLEY JF AND MACIOROWSKI Z. (1994). Clinical applications of DNA content parameters in patients with squamous cell carcinomas of the head and neck. *Semin. Oncol.*, **21**, 330–339.

ENSLEY J, CRISSMAN J, KISH J, JACOBS J, WEAVER A, KINZIE J, CUMMINGS G AND AL SARRAF M. (1986). The impact of conventional morphologic analysis on response rates and survival in patients with advanced head and neck cancers treated initially with cisplatin-containing combination chemotherapy. *Cancer*, **57**, 711–717.

ENSLEY JF, MACIOROWSKI Z AND PIETRASZKIEWICZ H. (1987). Tumor preparation for clinical application of flow cytometry. *Cytometry*, **8**, 488–493.

GASPARINI G, WEIDNER N, MALUTA S, PAZZA F, BORRACHI P, MEZZETTI M, TESTOLIN A AND BEVILACQUA P. (1993). Intratumoral microvessel density and p53 protein correlation with metastasis in head and neck squamous cell carcinoma. *Int. J. Cancer*, **55**, 739–744.

GREGG CM, BEALS TE, MCCLATCHY KM, FISHER SG AND WOLF GT. (1993). DNA content and tumor response to induction chemotherapy in patients with advanced laryngeal squamous cell carcinoma. *Otolaryngeal Head Neck Surg.*, **108**, 731–737.

JACOBSSON P, ENEROTH CM, KILLANDER D, MOBERGER G AND MARTENSSON B. (1973). Histological classification and grading of malignancy in carcinoma of the larynx. *Acta Radiol. Ther. Phys. Biol.*, **12**, 1–8.

KAPLAN EL AND MEIER P. (1958). Non parametric estimations from incomplete observations. *J. Am. Stat. Assoc.*, **53**, 457–481.

KLIJANIENKO J, MICHEAU C, CARLU C AND CAILLAUD JM. (1989). Significance of keratin 13 and 6 expression in normal, dysplastic and malignant squamous epithelium of pyriform fossa. *Virchows Arch. A Pathol. Anat.*, **416**, 121–124.

KLIJANIENKO J, EL-NAGGAR A, DE BRAUD F, MICHEAU C, JANOT F, LUBOINSKI B, GENTILE A, RUSSO A AND CVITKOVIC E. (1993). Keratins 6, 13 and 19 differential expression in squamous cell carcinoma of the head and neck. *Anal. Quant. Cytol. Histol.*, **15**, 335–340.

KLIJANIENKO J, EL-NAGGAR AK, DE BRAUD F, RODRIGUEZ-PERALTO JL, RODRIGUEZ R, ITZHAKI M, RUSSO A, JANOT F, LUBOINSKI B AND CVITKOVIC E. (1995). Tumor vascularisation, mitotic index, histopathologic grading and DNA ploidy in the assessment of 114 head and neck squamous cell carcinomas. *Cancer*, **75**, 1649–1656.

MANTEL N. (1966). Evaluation of survival data and two new rank order statistics arising in its consideration. *Cancer Chemother. Rep.*, **50**, 163–170.

MERKEL DE AND MCGUIRE WL. (1990). Ploidy, proliferative activity and prognosis. DNA flow cytometry of solid tumors. *Cancer*, **65**, 1194–1205.

NAKASHIMA T, MAEHARA Y, KOHNOE S, HAYASHI I AND KATSUTA Y. (1990). Histologic differentiation and chemosensitivity of human head and neck squamous cell carcinomas. *Head Neck*, **12**, 406–410.

ROLAND NJ, CASLIN AW, NASH J AND STELL PM. (1992). Value of grading squamous cell carcinoma of the head and neck. *Head Neck*, **14**, 224–229.

TUBIANA M. (1993). Kinetics of tumor cell proliferation. In *Handbook of Chemotherapy in Clinical Oncology*. Cvitkovic E, Droz JP, Armand JP, Khoury S (eds) 2nd ed pp. 23–35. Scientific Communication International: Paris.

VAN DER VELDEN LA, SCHAAFSMA HE, MANNI JJ, RAMAEKERS FC AND KUIJPERS W. (1993). Cytokeratin expression in normal and (pre) malignant head and neck epithelia: an overview. *Head Neck*, **15**, 133–146.

VAN HOEF MEHM, KNOX WF, DHESI SS, HOWELL A AND SCHOR AM. (1993). Assessment of tumor vascularity as a prognastic factor in lymph node negative invasive breast cancer. *Eur. J. Cancer*, **29A**, 1141–1145.

WALTER MA, PETERS GE AND PEIPER SC. (1991). Predicting radioresistance in early glottic squamous cell carcinoma by DNA content. *Ann. Othorhinolaryngol.*, **100**, 523–526.

WEIDNER N, SEMPLE JP, WELCH WR AND KOLKMAN J. (1991). Tumor angiogenesis. Correlation in invasive breast carcinoma. *N. Engl. J. Med.*, **324**, 1–8.

WORLD HEALTH ORGANIZATION (1978). Histological typing of upper respiratory tract tumors. In *International Histological Classification of Tumors*, no. 19, Shanmugaratnam K and Sobin LH (eds) p. 14. WHO: Geneva.

ZATTERSTROM UK, WENNERBERG J, EWERS SB, WILLEN R AND ATTEWELL R. (1991). Prognostic factors in head and neck cancer. Histologic grading, DNA ploidy, and nodal status. *Head Neck*, **13**, 477–487.